# Acute cholecystitis during COVID-19 pandemic: a multisocietary position statement

**DOI:** 10.1186/s13017-020-00317-0

**Published:** 2020-06-08

**Authors:** Fabio Cesare Campanile, Mauro Podda, Alberto Arezzo, Emanuele Botteri, Alberto Sartori, Mario Guerrieri, Elisa Cassinotti, Irnerio Muttillo, Marcello Pisano, Riccardo Brachet Contul, Giancarlo D’Ambrosio, Diego Cuccurullo, Carlo Bergamini, Marco Ettore Allaix, Valerio Caracino, Wanda Luisa Petz, Marco Milone, Gianfranco Silecchia, Gabriele Anania, Antonino Agrusa, Salomone Di Saverio, Salvatore Casarano, Caterina Cicala, Piero Narilli, Sara Federici, Massimo Carlini, Alessandro Paganini, Paolo Pietro Bianchi, Adelona Salaj, Andrea Mazzari, Roberto Luca Meniconi, Alessandro Puzziello, Giovanni Terrosu, Belinda De Simone, Federico Coccolini, Fausto Catena, Ferdinando Agresta

**Affiliations:** 1 Division of General Surgery, San Giovanni Decollato-Andosilla Hospital, Civita Castellana, ASL VT Italy; 2 Department of Surgery, Azienda Ospedaliero-Universitaria di Cagliari, Policlinico Universitario di Monserrato “Duilio Casula” University of Cagliari, SS 554, Km 4,500, Monserrato, 09042 Cagliari, Italy; 3 Department of Surgical Sciences, University of Torino, Turin, Italy; 4 General Surgery, ASST Spedali Civili di Brescia, Montichiari, Italy; 5 Department of General Surgery, Ospedale di Montebelluna, Montebelluna, Italy; 6 Department of General Surgery, Università Politecnica delle Marche, Ancona, Italy; 7 Chirurgia Generale, Fondazione IRCCS Ca’ Granda Ospedale Maggiore Policlinico, Milan, Italy; 8 Department of General and Emergency Surgery, Ospedale San Filippo Neri, Roma, Italy; 9 Department of General Surgery, Ospedale San Marcellino di Muravera, Cagliari, Italy; 10 Department of General and Emergency Surgery, Ospedale Regionale Umberto Parini, Aosta, Italy; 11 Department of General Surgery, Surgical Specialties and Organ Transplantation, Rome, Italy; 12 Department of General Surgery, Ospedali dei Colli Monaldi Hospital, Naples, Italy; 13 Emergency Surgery Unit, Careggi Hospital, Florence, Italy; 14 2° General Surgery, Hospital “Spirito Santo”, Pescara, Italy; 15 Department of Surgery, IEO, European Institute of Oncology IRCCS, Milan, Italy; 16 Department of Clinical Medicine and Surgery, Federico II University of Naples, Naples, Italy; 17 Department of Medico-Surgical Sciences and Biotechnologies, University La Sapienza of Rome, Latina, Italy; 18 Department of Morphology, Surgery and Experimental Medicine, University of Ferrara, Ferrara, Italy; 19 Department of General and Emergency Surgery, University of Palermo, Palermo, Italy; 20 Department of Surgery, University of Insubria, Varese, Italy; 21 Operating theatre, Pia Fondazione Panico Hospital, Tricase, Lecce, Italy; 22 Operating theatre, Fondazione Policlinico Gemelli IRCSS, Rome, Italy; 23 Division of General Surgery, Casa di Cura Nuova Itor, Rome, Italy; 24 Department of Surgery, Sant’Eugenio Hospital, Rome, Italy; 25 Department of Surgery “Paride Stefanini”, La Sapienza University of Rome, Rome, Italy; 26 Department of General and Minimally Invasive Surgery, Misericordia Hospital, Grosseto, Italy; 27 Department of Mini-Invasive and General Surgery, Cristo Re Hospital, Rome, Italy; 28 Department of Surgery, San Camillo-Forlanini Hospital, Rome, Italy; 29 Department of General Surgery, Salerno University Hospital, Salerno, Italy; 30 Department of General Surgery, Clinica Chirurgica, University of Udine, Udine, Italy; 31 Department of Visceral Surgery, Centre Hospitalier Intercommunal Poissy/Saint-Germain-en-Laye, Poissy, France; 32 Emergency Surgery Unit and Trauma Center, Pisa University Hospital, Pisa, Italy; 33 Emergency and Trauma Surgery, Maggiore Hospital, Parma, Italy; 34 Department of General Surgery, Ospedale Civile, Adria, Italy

**Keywords:** Acute cholecystitis, Emergency surgery, COVID-19 pandemic, New coronavirus, Position statement

## Abstract

Following the spread of the infection from the new SARS-CoV2 coronavirus in March 2020, several surgical societies have released their recommendations to manage the implications of the COVID-19 pandemic for the daily clinical practice. The recommendations on emergency surgery have fueled a debate among surgeons on an international level.

We maintain that laparoscopic cholecystectomy remains the treatment of choice for acute cholecystitis, even in the COVID-19 era. Moreover, since laparoscopic cholecystectomy is not more likely to spread the COVID-19 infection than open cholecystectomy, it must be organized in such a way as to be carried out safely even in the present situation, to guarantee the patient with the best outcomes that minimally invasive surgery has shown to have.

## Introduction

Following the spread of the infection from the new SARS-CoV2 coronavirus in March 2020, several surgical societies have released their recommendations to manage the implications of the COVID-19 pandemic for the daily clinical practice.

The recommendations on emergency surgery have fueled a debate among surgeons on an international level.

The SICE (Società Italiana di Chirurgia Endoscopica e Nuove Tecnologie), ACS-Italy Chapter (American College of Surgeons), AICO (Associazione Italiana infermieri di Camera Operatoria), CRSA (Clinical Robotic Surgery Association), SICG (Società Italiana di Chirurgia Geriatrica), SICOP (Società Italiana di Chirurgia dell’Ospedalità Privata), SPIGC (Società Polispecialistica Italiana dei Giovani Chirurghi), and the WSES (World Society of Emergency Surgery) have come out in favor of a rational analysis of the issue, especially about the choice of the surgical techniques to be implemented, preferring a “selective” approach that does not exclude the use of laparoscopy a priori but, instead, strongly considers it.

This approach is based on an analysis of the organization of human and logistical resources within which each of us operates, and takes into account the surgical skills that each surgeon has developed in the non-COVID era.

## Surgical societies and recommendations

The British Intercollegiate General Surgery Guidance on COVID-19 stated that during the COVID-19 pandemic, “whenever non-operative management is possible (such as for early appendicitis and acute cholecystitis), this should be implemented” [1].

Other surgical societies, however, including the SICE, the Society of American Gastrointestinal and Endoscopic Surgeons (SAGES), and the European Association for Endoscopic Surgery (EAES), have recommended a more patient-centered and hospital-centered approach [2–4].

There is still an essential ongoing debate on the specific question: “should we change our surgical indications for urgent conditions in this global situation?”

Reports from China told us that asymptomatic COVID-19-positive patients undergoing surgery go against unfavorable clinical outcomes, characterized by increased mortality and pulmonary complication rates [5].

This issue, along with the increased non-surgical care load that has impacted, and in some cases continues to impact profoundly on the activity of many hospitals in the world and also in Italy, has favored a change in the therapeutic approach for some surgical diseases, including acute cholecystitis (AC).

The debate that arose from these recommendations has revealed some further concerns about the possible evolution towards the aggravation of AC during non-operative treatment, such as to require a higher level of care following the failure of antibiotic therapy. A level of care that would not be possible to achieve in specific contexts with intensive care units still occupied by patients with COVID pneumonia.

As a scientific society, we must remember that therapeutic indications are established based on the best scientific evidence available at the moment and organizational choices must be founded on the evidence that science and research make available to health systems. This underlying assumption should never be forgotten, and even the possible revision of the surgical indications for the COVID-19 emergency (or other future emergencies) should take into account this fundamental principle.

Therefore, it is necessary to refer to the best available evidence to choose the therapeutic strategy for our patients, and do not allow the pressure imposed by the emergency conditioning to change our choices.

## What therapeutic strategy for acute cholecystitis during the COVID-19 pandemic?

Laparoscopic cholecystectomy (LC) remains the treatment of choice for AC, even in the COVID-19 era.

All current guidelines recommend LC as the gold standard of therapy for AC, because of the better results in terms of mortality, morbidity, and postoperative hospital stay compared to open cholecystectomy (OC) [6–8].

The Italian guidelines, promoted by SICE in 2012 in collaboration with all the leading Italian scientific societies [8] and our evidence-based guidelines on laparoscopic cholecystectomy published in 2015 [6], reiterated that “patients with acute cholecystitis should be treated with laparoscopic cholecystectomy” with a grade of recommendation A in the former and “strong” in the latter. This indication also applies in the case of elderly patients and those with severe AC. The guidelines from the World Society of Emergency Surgery (WSES) agree with this setting [7, 9].

Several studies have emphasized that many toxic components of the surgical smoke may endanger the operating team’s health.

Blood-borne viruses (HPV, HBV, HIV) are known to be present in the plume produced by electrocautery and other energy devices [10, 11]. However, although the SARS-Cov-2 RNA has recently been detected in the peritoneal cavity [12], there is no evidence to indicate the presence of SARS-CoV-2 in surgical smoke.

No evidence emerged suggesting that the risk of COVID-19 infection related to LC may be higher than that of OC, neither for the patient nor for the health professionals. Therefore, this working group does not consider that patients should be denied the benefits that high-quality studies have shown to be associated with LC. We recommend surgeons to take the necessary safety measures to reduce the risk of viral diffusion in the operating theater and ensure that patients continue to benefit from advantages of laparoscopic surgery [13].

If, on the one hand, laparoscopy contains the surgical smoke within the peritoneal cavity, on the other, the pneumoperitoneum evacuation could put the staff at risk of infection.

We suggest filtering the pneumoperitoneum through filters able to remove most viral particles. The ULPA (ultra-low particulate air) filters are extremely efficient to filter the SARS-CoV-2 virus whose diameter is about 0.06–0.14 μm. According to the ISO standard 29463 (issued to harmonize the European Standard EN 1822 and the US MIL-STD-282), an ULPA filter must have a ≥ 99.9995% efficiency at filtering particles with a MMPS (most penetrating particle size) of 0.12 μm. The MMPS is the particle that the filter is less efficient to remove. Smaller particles are filtered with an even higher efficiency. The use of these filters is recommended [3, 4, 13].

It is crucial nowadays to examine the evidence concerning the timing of LC for AC, which compares the results of “early” cholecystectomy with those of “delayed” cholecystectomy, that is carried out after a period of conservative therapy to overcome the acute phase.

Early cholecystectomy is recommended in all the guidelines mentioned above, based on the results of several meta-analyses of randomized controlled trials that compared the two different approaches. It has been demonstrated that early cholecystectomy (i.e., performed “as soon as possible” after the onset of symptoms and, in any case, not later than the tenth day from it) has not shown inferior results compared with the delayed one in terms of morbidity, mortality, and conversion rate (i.e., six weeks after the acute episode).

Therefore, early cholecystectomy is preferable to the delayed, for its shorter overall length of hospitalization (considering the sum of the stay of the first hospitalization—that is, of acute cholecystitis—and the second—that of the delayed intervention).

The equivalency of the two strategies in terms of morbidity, mortality, and conversion rate cannot justify the systematic use of delayed cholecystectomy. During the COVID epidemic, it may instead be desirable to postpone the surgical act away from the epidemic period, even at the cost of greater use of resources of the health system (length of stay).

The equivalency in terms of morbidity and mortality between the two approaches can be a solid basis for more extensive use of delayed cholecystectomy, based on the analysis of the human and logistical resources of the hospital in which each of us works, the organizational pathways adopted, and the local epidemiological situation.

It is mandatory that during the conservative treatment period, attention must be paid to monitoring sepsis parameters and pain progression despite appropriate analgesic therapy. The danger of progress of the septic state and the risk of progression towards the gangrene, emphysematous cholecystitis, or the rupture of the gallbladder may, anyway, require emergency cholecystectomy.

If it is true that in the pre-COVID period, cholecystectomy in patients classified as high risk according to the various guidelines has a mortality rate that can reach 19% [14], clearly this aspect assumes greater relevance in positive or suspected COVID-19 patients, which are considered at high surgical risk in themselves.

Both the incidence of AC and the mortality from COVID-19 are higher in elderly patients. Although elderly patients are more likely to present with different comorbidities that complicate any postoperative course, early LA for AC is safe and effective in this group of patients, albeit burdened by increasing conversion rates [15].

The Italian guidelines (by SICE, ACOI, SIC, SICUT, SICOP) on LC [6] and the recent WSES guidelines [7, 9] refer as in the case of patients with prohibitive surgical risk (“unfit for surgery”) percutaneous drainage of the gallbladder may be considered after the failure of conservative therapy with antibiotics. However, it must be stressed that advanced age, or other factors of higher COVID-19 risk, cannot be regarded as sufficient to indicate this alternative treatment except in real conditions of the impracticability of cholecystectomy.

The analysis of the international literature, despite being mainly based on observational studies with a low level of evidence, demonstrates a high mortality rate for patients undergoing percutaneous gallbladder drainage. High mortality was also shown in recent extensive retrospective analyzes [16, 17].

Moreover, the CHOCOLATE trial, a randomized controlled trial that had been started to compare the results of percutaneous drainage vs cholecystectomy, was prematurely terminated because the ethical problems arising from the observation of the high mortality in patients who underwent percutaneous drainage did not allow the further continuation of the study [18].

As indicated, the execution of a percutaneous cholecystostomy (only in patients with prohibitive surgical risk) takes place, as specified above, after the failure of conservative therapy, which constitutes the first therapeutic strategy in these particularly fragile patients.

Of all the options currently listed in the literature (percutaneous transhepatic cholecystostomy, transpapillary drainage, transmural drainage), percutaneous transhepatic cholecystostomy is generally the preferred one, due to its simplicity of execution, safety, and reduced costs.

The optimal timing for performing percutaneous cholecystostomy is widely debated. However, when the cholecystostomy is carried out within 24 h from the onset of the clinical presentation is associated with fewer complications in terms of bleeding and lower hospital stay [19]. However, the timing of percutaneous cholecystostomy depends primarily on the clinical indication. Urgent drainage should be considered in case of severe sepsis in a patient not eligible for surgery.

For the remaining patients not eligible for surgery, it is common practice to proceed with cholecystostomy if the patient does not improve within 1–3 days of starting antibiotic therapy.

## Conclusions

We maintain that, since laparoscopy is not more likely to spread the COVID-19 infection than open surgery, it must be organized in such a way as to be carried out safely even in the present situation, to guarantee the patient with the best outcomes that minimally invasive surgery has shown to have. In the case of patients unfit for surgery, percutaneous transhepatic cholecystostomy may be considered after the failure of conservative therapy with antibiotics.

## Data Availability

There are no individual author data that reach the criteria for availability.
